# Hypertonic saline infusion does not improve the chance of primary fascial closure after damage control laparotomy: a randomized controlled trial

**DOI:** 10.1186/s13017-023-00475-x

**Published:** 2023-01-09

**Authors:** Alberto F. García, Ramiro Manzano-Nunez, Diana Cristina Carrillo, Julian Chica-Yanten, María Paula Naranjo, Álvaro I. Sánchez, Jorge Humberto Mejía, Gustavo Adolfo Ospina-Tascón, Carlos A. Ordoñez, Juan Gabriel Bayona, Juan Carlos Puyana

**Affiliations:** 1 Department of Surgery, Fundación Valle del Lili, Cali, Colombia; 2 Department of Intensive Care, Fundación Valle del Lili , Cali, Colombia; 3 Clinical Research Center, Fundación Valle del Lili , Cali, Colombia; 4 Department of Surgery, School of Medicine, Universidad del Valle, Cali, Colombia; 5 Vall d’Hebron Institute of Research, Barcelona, Spain; 6 Vall d’Hebron Hospital Universitari, Barcelona, Spain; 7 Universitat Autònoma de Barcelona, Barcelona, Spain; 8 Professor of Surgery Director Global Health, Critical Care and Clinical Translational Surgery, University of Pittsburgh, Pittsburgh, PA USA; 9 Translational Research Laboratory in Critical Care Medicine (TransLab-CCM), Universidad Icesi, Cali, Colombia; 10 Department of Surgery, Universidad Javeriana, Bogotá, Colombia; 11 Present Address: Department of Surgery, Universidad Sanitas, Bogotá, Colombia

**Keywords:** Damage control surgery, Abdominal injuries, Hypertonic saline, Trauma and injuries

## Abstract

**Background:**

Previous observational studies showed higher rates of abdominal wall closure with the use of hypertonic saline in trauma patients with abdominal injuries. However, no randomized controlled trials have been performed on this matter. This double-blind randomized clinical trial assessed the effect of 3% hypertonic saline (HS) solution on primary fascial closure and the timing of abdominal wall closure among patients who underwent damage control laparotomy for bleeding control.

**Methods:**

Double-blind randomized clinical trial. Patients with abdominal injuries requiring damage control laparotomy (DCL) were randomly allocated to receive a 72-h infusion (rate: 50 mL/h) of 3% HS or 0.9 N isotonic saline (NS) after the index DCL. The primary endpoint was the proportion of patients with abdominal wall closure in the first seven days after the index DCL.

**Results:**

The study was suspended in the first interim analysis because of futility. A total of 52 patients were included. Of these, 27 and 25 were randomly allocated to NS and HS, respectively. There were no significant differences in the rates of abdominal wall closure between groups (HS: 19 [79.2%] vs. NS: 17 [70.8%]; *p* = 0.71). In contrast, significantly higher hypernatremia rates were observed in the HS group (HS: 11 [44%] vs. NS: 1 [3.7%]; *p* < 0.001).

**Conclusion:**

This double-blind randomized clinical trial showed no benefit of HS solution in primary fascial closure rates. Patients randomized to HS had higher sodium concentrations after the first day and were more likely to present hypernatremia. We do not recommend using HS in patients undergoing damage control laparotomy.

*Trial registration* The trial protocol was registered in clinicaltrials.gov (identifier: NCT02542241).

## Introduction

Since patients with abdominal trauma often present severe injuries and impaired physiology, they tend to require damage-control abdominal surgery followed by damage-control resuscitation [1, 2]. Trauma surgeons treating these patients prioritize bleeding and contamination control and leave the abdomen open during surgery [3]. Then, patients are transferred to the ICU for appropriate monitoring and hemostatic resuscitation. One of the objectives of the damage-control approach is to prioritize physiology stabilization before definitive injury repair and abdominal wall closure. However, trauma surgeons often face the trade-off between leaving the abdominal wall open beyond the necessary time and the complications that can arise from an open abdomen. Therefore, interventions to enhance resuscitation while simultaneously reducing the time in which an open abdomen is required, improving the chance of achieving early abdominal wall closure, are needed.

A potential intervention to reduce the volume of intravenous fluids infused without putting resuscitation in peril is hypertonic saline (HS) infusion [4, 5]. Prior observational studies have shown improved outcomes [6–8], including higher rates of abdominal wall closure with reduced times to it with the use of HS during the resuscitation of injured patients with abdominal trauma requiring damage control surgery with an open abdomen. However, these studies are observational, retrospective, and prone to bias. Thus, comprising the validity and clinical applicability of their results. Here, we present a double-blind, randomized clinical trial to assess the effect of 3% hypertonic saline solution on primary fascial closure and the timing of abdominal wall closure among patients who underwent damage control laparotomy (DCL) for bleeding control.

## Methods

### Setting, study design, and oversight

This double-blind, randomized, placebo-controlled trial was conducted at Fundación Valle del Lili (FVL) University hospital between November 2015 and August 2018. The FVL is a fourth-level university hospital equivalent to a US-level I trauma center, and it has 523 beds. Of these, 205 are ICU beds and 10 are reserved for trauma patients. The FVL trauma center admits approximately 700 moderate-to-severe trauma patients per year, serving as one of the largest trauma referral centers in the southwest region of Colombia.

The trial protocol was registered in clinicaltrials.gov (identifier: NCT02542241). The protocol was designed by the Trial Steering Committee and was reviewed and approved by the FVL ethical and biomedical research committee. Written informed consent was obtained from all patients or another surrogate decision-maker. Consent by an independent physician and deferred consent was used as a surrogate to subject consent when appropriate. In these cases, the trial team performed the randomization following pre-specified trial protocol criteria and then requested for the patient’s or representative’s (proxy) informed consent in a later phase.

An independent committee and the staff from the FVL clinical research center regularly monitored the trial to check for protocol compliance and data transparency.

### Patients

Adult patients with traumatic injuries were eligible for the study if they were to undergo damage control abdominal surgery in the index laparotomy. Specific inclusion criteria were: 1. informed consent obtained before any trial-related activities, 2. age above or equal to 18 years at the time of inclusion, and 3. patients with blunt or penetrating trauma and requiring DCL for abdominal injuries. The decision to perform an emergent laparotomy was taken by the attending trauma surgeon at the emergency department in patients with signs of exsanguination/hemodynamic instability, acute abdomen or evisceration. When in the operating room, patients underwent a DCL (involving surgical control of bleeding and contamination plus packing with laparotomy pads plus leaving the abdominal wall open) if there was evidence of hypothermia, metabolic acidosis or hyperlactatemia (reflecting physiological exhaustion), hemoperitoneum and/or destructive intra-abdominal organ injuries. A detailed description on how we approach and provide surgical treatment to patients with abdominal injuries requiring damage-control surgery at our institution is described elsewhere [9, 10].

### Exclusion criteria

Patients were excluded if the time between the occurrence of his/her traumatic injuries and the randomization was longer than four hours, had a severe traumatic brain injury, and had a high probability of death in the first 48 h. Although the treating surgeon subjectively determined this, the decision was based on what is known about the epidemiology of deaths after trauma [11–13]. Therefore, patients with a high probability of death during the first 48 h were those with destructive lesions associated with major exanguination leading to refractory shock. For example, patients with abdominal injuries and concomitant/coexistent high grade ≥ 4 solid organ injuries, severe pulmonary injuries causing massive hemothorax, heart injuries, or single/multiple injuries to major named axial torso vessels.

Patients were also excluded if the indications for performing a DCL were different from the initial trauma (i.e., DCL in the context of post-traumatic intrabdominal infections, patients in whom the abdomen was closed at the index surgery but required an emergent relaparotomy for postoperative bleeding or control of contamination). Pregnant women were also excluded.

### Study protocol

Patients in both study groups received trauma care in the emergency department and the operating group following current clinical practice guidelines and institutional protocols. After the decision to perform a DCL, patients underwent randomization in a double-blind manner. Randomization was performed in permuted blocks of four and six by an independent statistician using Random Allocation Software® [14]. The assignment was concealed until consent was given. Then, the investigator confirmed the inclusion in an internet-based platform, which revealed the allocation after confirming the consent and the inclusion criteria. Regularly, randomization happened immediately after the index surgery when preparing for the transfer to the ICU or in the first minutes in the ICU.

According to their allocation, the patients received a 72-h infusion (rate: 50 mL/h) of 3 N hypertonic saline or 0.9 N isotonic saline after the index damage control laparotomy. The infusion was administered in the intensive care unit. The presentation of the study solutions was similar. Neither the treating group nor the patients nor the investigators could discern their composition. The treating team made decisions regarding other fluids, blood components, vasopressors, inotropic support, or monitoring according to the usual practice. The study infusion was considered at all times when planning and executing fluid resuscitation. Double-blind was achieved by the use of similar appearing saline bags in the two groups.

Decisions regarding the timing of the reoperations and the abdominal closure were made by the surgical team according to the need for reconstruction of specific lesions, the occurrence of complications, and the fluid balance. In each patient, the surgeons worked to reconstruct the lesions and close the abdomen as early as possible.

The protocol for organ injury management and abdominal wall closure has been previously published [9, 10, 15]. In brief, delayed abdominal closure was considered a bridge to achieve three main goals: 1. compartment syndrome prevention, 2. definitive control of bleeding and contamination, and 3. definitive wound healing. Therefore, the first reoperation was performed as early as possible after correcting the patient’s physiological derangement (control of acidosis, hypothermia, and coagulopathy). It is our surgical practice to perform definitive abdominal wall closure when: all the injuries are correctly managed, the patient physiology is stabilized, and there is no evidence of abdominal contamination focuses/absence of intra-abdominal infections. Also, definitive closure is performed when the abdominal cavity can be closed without tension. Other factors that the surgeon considers to perform definitive closure are the fluid balance, the intestinal edema found at surgery, and the change in the airway pressure or the tidal volume during the procedure.

### Study end points and definitions

The primary endpoint of the trial was the proportion of patients with abdominal wall closure in the first seven days after the index surgery.

Secondary endpoints were: 28-day mortality, the fluid balance during the first 72 h, the proportion of patients requiring reoperation for intra-abdominal hypertension, the proportion of patients with abdominal compartment syndrome (defined as sustained intra-abdominal pressure > 20 mm Hg and a new organic dysfunction), the proportion of patients with organic dysfunction, defined as a Sequential Organ Failure Assessment (SOFA score) > 2 [16], the proportion of patients with ARDS [17], and the proportion of patients with hypernatremia.

### Statistical analysis

The trial was designed on the assumption of the superiority principle to have 80% power with a *p* < 0.05 to detect a 60-to-75% difference in the probability of closing the abdomen on the seventh day after the index laparotomy with the therapy with hypertonic saline. With the addition of 20% for losses, we calculated 200 patients needed in each study group.

The trial team had full access to the data and was responsible for the analysis. All analyses were performed in a blinded manner so that investigators could not know if subjects were assigned to the control or treatment group.

Statistical analyses were performed on Stata 15.1® (College Station, Tx). Categorical variables are presented as frequencies and percentages. The normality of continuous variables was examined by the Shapiro–Wilk test. Afterward, they are presented as mean and standard deviation or median and interquartile rank.

Categorical variables were compared with Chi^2^ or Fisher’s Exact Test, as indicated. Continuous with Student’s *T* test or Wilcoxon–Mann–Whitney according to normality. Time-to-event variables were presented as Kaplan–Meier curves and compared with the Logrank test.

Interim analyses were planned at enrollment of 50, 100, 200, and 300 patients, with predefined termination criteria for futility or effectivity [18]. We chose the Haybittle-Peto criteria [19, 20] for stopping for effectivity and the analysis of conditional power for stopping for futility [21, 22].

## Results

**Fig. 1:**
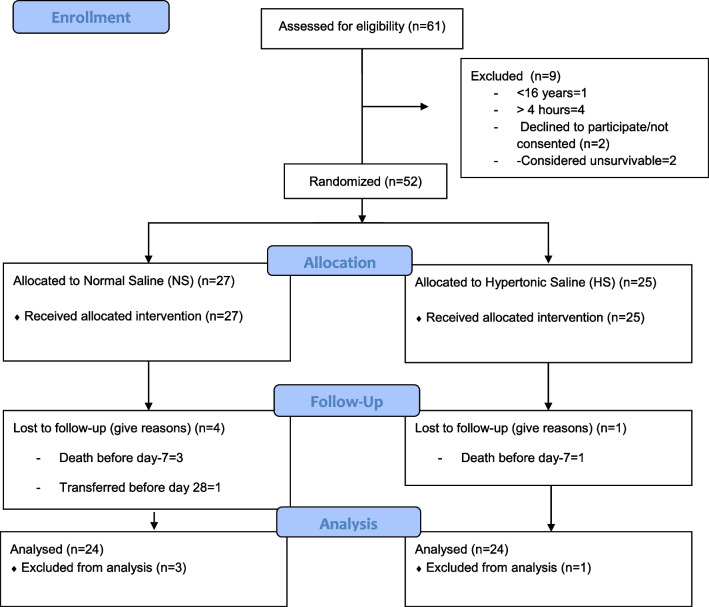
Consort flow diagram

The study was suspended in the first interim analysis because of futility. Of the 61 screened patients, nine were excluded. The most frequent cause was the notification to the investigation group after the 4-h limit. Figure 1 shows the CONSORT flow diagram for the present randomized trial.

A total of 52 patients were included, and 44 (84.6%) were male. The trauma mechanism was penetrating in 41 cases (78.9%). The physiologic derangement was moderate at admission, with a median interquartile range (IQR) of RTS of 7.11 (6.08–7.84) (Table 1). The median interquartile range (IQR) of systolic blood pressure, respiratory rate, and Glasgow coma scale were 90 (20–122) mm Hg, 23 (19–29.5), and 14 (12.5–15), respectively. (Table 1).

**Table 1:** Hypertonic saline after a damage control laparotomy. Randomized controlled clinical trial. Baseline information

Variable	Total	NS	HS
Patients, *n* (%)	52	27	25
Age, mean (SD)	35.1(± 14.1)	32.9 (± 13.9)	37.5 (± 14.1)
Male, *n* (%)	44 (84.6%)	22 (81.5%)	22 (88.0%)
Trauma mechanism			
Penetrating, *n* (%)	41 (78.9%)	24 (88.9%)	17 (68.0%)
Blunt, *n* (%)	11 (21.1%)	3 (11.1%)	8 (32.0%)
Systolic BP, mm Hg, median (IQR)	90 (60–122)	80 (60–129)	91 (60–112)
Respiratory rate, breaths/min, median (IQR)	23 (19–29.5)	22 (19–30)	24 (19–28)
Glasgow Coma Score, median (IQR)	14 (12.5–15)	15 (10–15)	14 (13–15)
RTSt, median (IQR)	11 (9 − 12)	11 (9 −12)	11 (10 −12)
RTSs, median (IQR)	7.11 (6.08–7.84)	7.11 (5.78–7.84)	7.11 (6.38–7.84)
AIS thorax, median (IQR)	0 (0 -3)	0 (0–3)	0 (0–3)
AIS abdomen, median (IQR)	4 (3.5–5)	4 (3.5–5)	4 (3–5)
ISS, median (IQR)	26 (17.5–35)	26 (17–35)	29 (25–41)
ATI, median (IQR)	25.5 (12.5–35.5)	26 (13–37)	20 (12–35)
Abdominal damage control			
Liver, n (%) Major vascular, *n* (%) Hollow viscus, *n* (%) Other, *n* (%)	25 (48.1%) 39 (75.0%) 33 (63.5%) 38 (73.1%)	13 (48.1%) 20 (74.1%) 17 (63.0%) 23 (85.2%)	12 (48.0%) 19 (76.0%) 16 (64.0%) 15 (60.0%)
Extraabdominal damage control, *n* (%)	46 (88.5%)	25 (92.6%)	21 (84.0%)
Abdominal closure			
Commercial, *n* (%) Barker, *n* (%)	47 (90.4%) 5 (9.6%)	25 (92.6%) 2 (7.4%)	22 (88.0%) 3 (12.0%)
PRBC, first 6 h, median (IQR)	4 (2–6)	4 (3–6)	4 (2–6)

*NS* normal saline; *HS* hypertonic saline; *SD* standard deviation; *IQR* interquartile range; *RTSt* revised trauma score, used for triage; *RTSt* revised trauma score, used for survival calculation; *ISS* injury severity score; *ATI* abdominal trauma index; *PRBC* packed red blood cells

The anatomic severity of injuries, measured by the ISS [23] and ATI [24] scores, was classified as severe in more than 50% of the cases and critical in about one-fourth (Table 1). The most frequently compromised structure was the major vessels in the abdomen (75%), followed by the hollow viscus (63.5%) and the liver (48%) (Table 1).

In 88.5% of the cases, an additional damage control procedure was required for the repair of an injury outside the abdomen. The abdominal cavity was transiently closed with a negative pressure-assisted system in all patients. A commercial device was used in 90.4% of the patients (Table 1).

Twenty-seven patients were randomly assigned to normal saline (NS) and 25 to 3 N hypertonic saline (HS) (Fig. 1). The comparison between the groups did not show differences in the demographic information, the trauma mechanism or severity, the injured organs, the associated trauma, the technique used for the abdominal wall closure, or the transfusions required (Table 1).

Of the 27 patients randomized to the NS group, three died before the 7th day and could not be analyzed for the primary endpoint, and one was sent to another hospital on day eight and could not be analyzed for 28-day mortality. Of the 25 subjects assigned to the HS group, one died in the first week and could not be analyzed for the primary outcome.

**Fig. 2:**
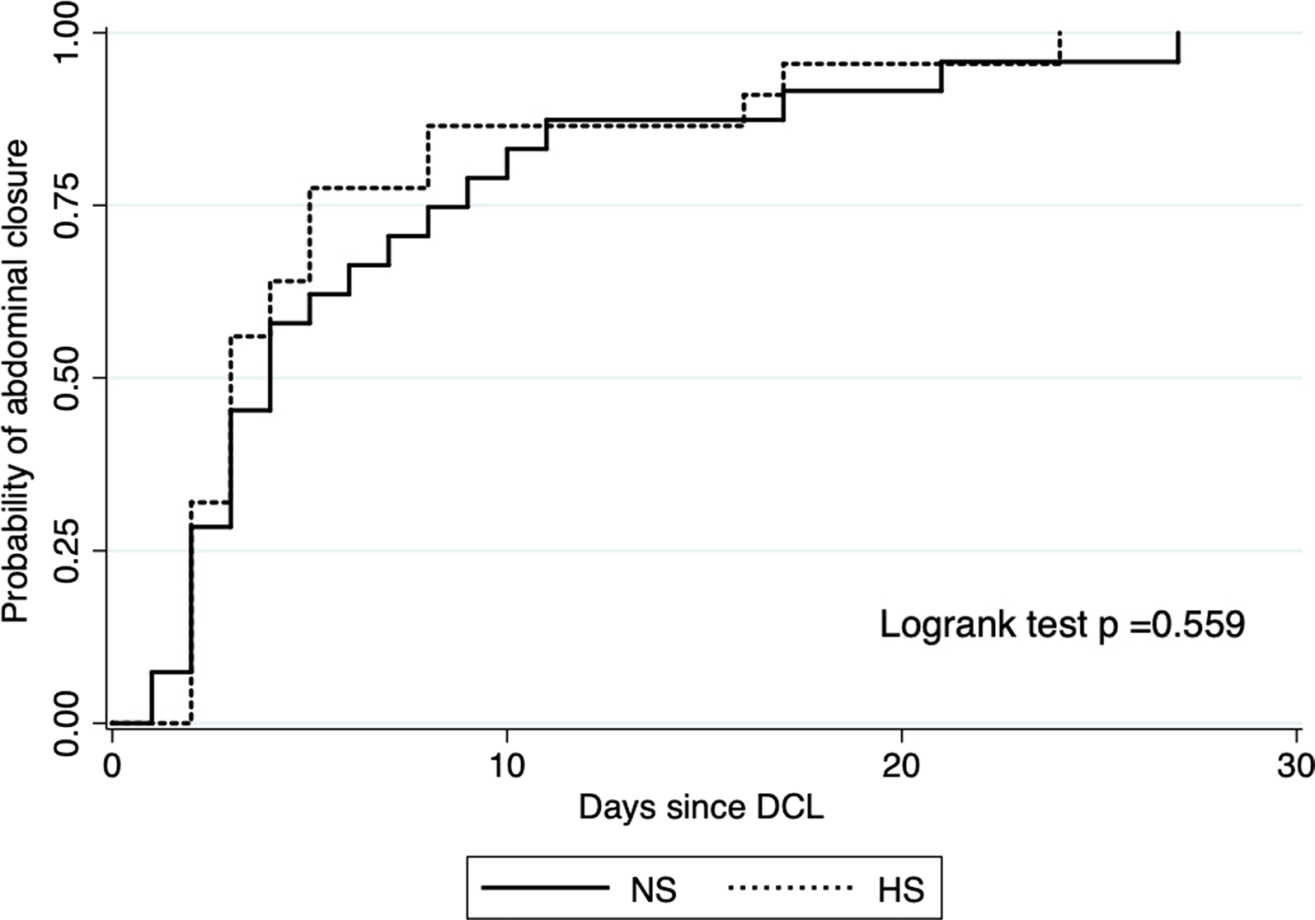
Kaplan–Meier curve for the probability of abdominal wall closure over time

Of the 24 patients on the NS analyzed for abdominal closure, 17 (70.8%) were closed during the first seven days. Of the 24 on the HS, 19 (79.2%) were closed in the same period. This difference was not significant (*p* = 0.71) (Table 2). Survival analysis confirmed the absence of difference between groups (*p* = 0.559) (Fig. 2).

**Table 2:** Hypertonic saline after a damage control laparotomy. Randomized controlled clinical trial. Outcomes

Outcome	NS	HS	p
Abdominal closure in the first 7 days, n (%)	17/24 (70.8%)	19/24 (79.2%)	0.71^1^
Day 28 mortality, *n* (%)	3/26 (11.5%)	2/24 (8.0%)	1.00^2^
Abdominal compartment syndrome, *n* (%)	0 (–)	2 (8.0%)	0.27^2^
Total SOFA score	5 (7.5–9)	6 (4–14)	0.95^3^
Number of subjects with SOFA score > 6, *n* (%)	13 (59.1%)	12 (48.0%)	0.32^1^
ICU-free days in the first 30 days, median (IQR)	16 (1–23)	19 (11–24)	0.57^3^
Fluid balance			
Day 1, c.c., median (IQR)	2165 (1180–4119)	1828 (427–3344)	0.21^3^
Day 2, c.c., median (IQR)	678 (106–1599)	387 (− 125–1165)	0.58^3^
Day 3, c.c., median (IQR)	261 (− 32–950)	170 (− 136–580)	0.43^3^
First 72 h, c.c., median (IQR)	3957 (1767–5946)	2486 (650–5578)	0.29^3^

*NS* normal saline *HS* hypertonic saline *IQR* interquartile range

^1^Chi^2^

^2^Fisher’s Exact test

^3^Wilcoxon–Mann–Whitney

As shown in Table 2, 28-day mortality was 11.5% in the NS group and 8.5% in the HS group (*p* = 1.00). The number of patients with a SOFA score > 6 and the SOFA values were similar among both groups (p 0.32 and 0.95, respectively) (Table 2). Five patients in each group experienced an increase in serum creatinine (0.17). Positive balance during the first 72 h occurred in both groups and was slightly higher in the NS group (0.29) (Table 3).

The serum concentration of sodium was higher in the HS during the study. Table 2 shows the peak concentration on day 3 which was 141 versus 157, *p* < 0.001. The rate of hypernatremia was significantly higher in the HS group. While one episode of hypernatremia (3.7%) occurred in the group of NS, 11 (44%) were observed in the group of HS *p* < 0.001. Similarly, serum osmolality on day three was higher in the HS group (Table 3). The median (IQR) of the number of ICU-free days was 16 (1–23) in the NS group and 19 (11–24) in the HS group (*p* = 0.57).

**Table 3:** Hypertonic saline after a damage control laparotomy. Randomized controlled clinical trial. Security items

Variable	NS	HS	p
Fluid balance			
Day 1	2665.5 (678–261)	1828 (427–3344)	0.21^3^
Day 2	768 (106–1599)	387 (− 125–1165)	0.58^3^
Day 3	261 (− 32–950)	170 (− 136–580)	0.43^3^
Day 4	357 (− 280–729)	10 (− 811–557)	0.19^3^
Day 5	51.5 (− 1835–697)	133 (− 713–1101)	0.37^3^
Day 6	− 385 (− 1057–140)	60 (− 1200–580)	0.37^3^
Day 7	− 110 (− 1109–597)	267 (− 650–810)	0.68^3^
First 72 h	3957 (1767–5976)	2486 (650–5578)	0.29^3^
First 7 days	2998 (1278–6964)	3133 (− 1700–6603)	0.49^3^
Sodium concentration			
Day 0	140 (138–143)	141 (139–142)	–
Day 1	140 (138.5–142)	148 (144.5–151)	< 0.001^3^
Day 2	140 (138–143)	153 (150–157)	< 0.001^3^
Day 3	141 (139–144)	157 (149–161)	< 0.001^3^
Day 4	143 (140–146)	156 (146–160)	< 0.001^3^
Day 5	143.5 (140–147)	151 (144–156.5)	0.006^3^
Day 6	144 (139–147)	149 (144–155)	0.031^3^
Day 7	144 (140–147)	143 (140–147)	0.95^3^
Chloride concentration			
Day 0	105 (104–106)	106 (102–110)	0.65^3^
Day 1	105 (103–107)	114.5 (110.5–120.5)	< 0.001^3^
Day 2	107 (104.5–108.5)	120 (116–128)	< 0.001^3^
Day 3	106.5 (105–111)	124 (111–127)	< 0.001^3^
Day 4	107.5 (104–112)	122 (111–127)	< 0.001^3^
Day 5	106 (103–110)	115 (108–122)	0.008^3^
Day 6	108 (104–113)	113 (107–117)	0.08^3^
Day 7	107 (105–112)	108.5 (104–113)	0.58^3^
Osmolality			
Day 0	299 (297–306)	306 (300–316)	0.03^3^
Day 3	297.5 (290–305)	324 (308–333)	0.001^3^
Number of subjects with SOFA score > 6, n (%)	13 (59.1%)	12 (48.0%)	0.32^1^
RIFLE score			
Serum creatinine increase > 50%	3	2	0.17^2^
Serum creatinine increase > 100%	2	0	
Serum creatinine increase > 200%	0	3	
ICU-free days in the first 30 days, median (IQR)	16 (1–23)	19 (11–24)	0.57^3^

*NS* normal saline; *HS* hypertonic saline; *IQR* interquartile range

^1^Chi^2^

^2^Fisher’s exact test

^3^Wilcoxon–Mann–Whitney

### Early termination of the clinical trial

After recruiting the first 50 cases, an independent statistician performed the first interim analysis. A power of 0.09 was found, and a conditional power of 0.34 was calculated for the planned sample size. The 95% CI for the RR at this level would have been 0.99–1.27. The recalculation of the sample size at the same p and power, with the observed parameters, resulted in 1102 patients. With this information and the observation of a high proportion of patients developing hypernatremia in one of the groups, the data and safety monitoring board recommended stopping the trial.

## Discussion

In this randomized clinical trial, which was stopped early for futility, hypertonic saline neither improved primary fascial closure rates nor the time to it after DCL; however, it did increase the sodium concentrations and was associated with a higher rate of hypernatremia. This is, to our knowledge, the first randomized trial assessing the effect of infusing hypertonic saline solution on injured patients undergoing damage control laparotomy. The findings contradict previous observational studies that posed hypertonic saline solution as an intervention associated with higher fascial closure rates in DCL patients [6–8].

In line with the present results, the existing body of research on hypertonic solutions for trauma resuscitation has not been able to find survival benefits or consistent beneficial effects with the use of hypertonic saline in injured patients. Moreover, such as in this trial, previous resuscitation studies assessing the use of hypertonic solutions in trauma patients were halted due to futility [25–27]. For example, two previous large trials funded by the National Heart, Lung, and Blood Institute (NHLBI) [25–27], investigating the effect of HS administered during the prehospital resuscitation were stopped early because interim analyses demonstrated insufficient promise of an HS treatment benefit. Of concern, in one of these trials, an increase in mortality was found with HS (Table 4).

**Table 4:** Hypertonic saline after a damage control laparotomy. Randomized controlled clinical trial. Episodes of hypernatremia >159 Meq/Ll

Variable	NS	HS
During the infusion	0	10
After the infusion	1	1
Total	1	11

*NS* normal saline *HS* hypertonic saline

A relatively small body of literature is concerned with the safety and effectiveness of HS after emergent laparotomy. A seminal study in this area is the work of Harvin et al. [8], which showed that using 3% HS increased the fascial closure rate and decreased the time to fascial closure compared to the standard saline solution (early primary fascial closure: 78% in the NS group vs. 100% in the HS group, *p* = 0.01). Building upon this study, Loftus et al. [6, 7] published two studies that reported higher fascial closure rates with implementing a treatment bundle (including 3%-HS) for trauma patients requiring DCL. In these studies, patients resuscitated with 3%-HS were more likely to achieve early fascial closure; thus, providing evidence to underpin the implementation of trauma resuscitation protocols including 3%-HS as a smooth path to achieve fascial closure in patients undergoing DCL. The present randomized controlled trial results challenge previous observational studies and should be used as a source to develop clinical recommendations and implement changes in surgical practice.

In the present trial, patients randomized to 3% HS had a significantly higher sodium concentrations and were more likely to present hypernatremia. These findings are broadly consistent with the work of other studies in this area linking hypertonic saline infusion with hypernatremia. For example, Loftus et al. [6, 7] showed that among patients undergoing emergent laparotomy and temporary abdominal closure, those resuscitated with hypertonic saline had a significantly higher sodium concentration at 48 h following index laparotomy.

The findings presented in this randomized controlled trial do not support the recommendation to use hypertonic saline solution to achieve a favorable outcome regarding abdominal fascial closure. Moreover, given the evident uncertainty of benefits with the accompanying peril of acute dysnatremias, which in turn can lead to serious adverse events, and the well-known documented harms that the use of HS poses to trauma patients [5], we do not recommend its use in the setting of injured patients undergoing damage-control laparotomy.

Although this trial did not show positive results favoring HS, it might be possible to use it as a part of a bundle in future investigations. For example, in combination with a mesh-mediating traction system, which (by itself) has shown promising results, allowing a safe early abdominal closure [28]. For example, Alsaadi et al. [29] reported a series of 24 patients requiring emergency laparotomy in whom prophylactic onlay mesh was used. They found that this intervention was associated with acceptable wound-healing outcomes and recommended it as a potential intervention to prevent fascial dehiscence in injured patients.

More broadly, research is also needed to determine the role and effects of direct peritoneal resuscitation on post-DCL outcomes. To date, studies on direct peritoneal resuscitation have been performed primarily on animals [30], and little is known about its effect on clinically relevant outcomes. One randomized trial [31], including 52 patients undergoing damage control surgery, found that patients randomized to peritoneal resuscitation had higher rates of abdominal wall closure with reduced times to it, and lower rates of intra-abdominal infections. Therefore, if the debate about DCL and post-op care after abdominal trauma is to be moved forward, a better understanding of bundles, including direct peritoneal resuscitation and mesh traction systems (through well-designed clinical studies), needs to be developed [28].

## Limitations

Our study has limitations. First, the small number of patients available for analysis limits the results’ generalizability despite the randomization. Second, one of the most obvious shortcomings of this study is its potentially limited external validity derived from its single-center nature. Third, related to its low sample size, the trial certainly had a likelihood of type II error (declaring no differences between groups when, in fact, these differences exist). Thus, it could be argued that the study lacks sufficient power and should be interpreted as presenting inconclusive findings. However, we have presented the steps we followed to stop the trial, suggesting that completing the sample size would not have guaranteed reaching statistical significance.

## Conclusion

This randomized clinical trial showed no benefit of hypertonic saline solution in primary fascial closure rates. Patients randomized to HS had higher sodium concentrations after the first day and were more likely to present hypernatremia. We do not recommend using HS in patients undergoing damage control laparotomy.

## Data Availability

The datasets generated and/or analyzed during the current study are not publicly available due to privacy concerns but are available from the corresponding author on reasonable request.

## Abbreviations

DCL: Damage control laparotomy
HS: Hypertonic saline
NS: Normal saline
